# Microbial Community Metabolic Modeling: A Community Data‐Driven Network Reconstruction

**DOI:** 10.1002/jcp.25428

**Published:** 2016-06-02

**Authors:** Christopher S. Henry, Hans C. Bernstein, Pamela Weisenhorn, Ronald C. Taylor, Joon‐Yong Lee, Jeremy Zucker, Hyun‐Seob Song

**Affiliations:** ^1^ Division of Mathematics and Computer ScienceArgonne National LaboratoryArgonneIllinois; ^2^ Computation InstituteUniversity of ChicagoChicagoIllinois; ^3^ Biodetection SciencesNational Security DirectoratePacific Northwest National LaboratoryRichlandWashington; ^4^ Biological Sciences DivisionEarth and Biological Sciences DirectoratePacific Northwest National LaboratoryRichlandWashington; ^5^ The Gene and Linda Voiland School of Chemical Engineering and BioengineeringWashington State UniversityPullmanWashington; ^6^ Division of BiosciencesArgonne National LaboratoryArgonneIllinois

## Abstract

Metabolic network modeling of microbial communities provides an in‐depth understanding of community‐wide metabolic and regulatory processes. Compared to single organism analyses, community metabolic network modeling is more complex because it needs to account for interspecies interactions. To date, most approaches focus on reconstruction of high‐quality individual networks so that, when combined, they can predict community behaviors as a result of interspecies interactions. However, this conventional method becomes ineffective for communities whose members are not well characterized and cannot be experimentally interrogated in isolation. Here, we tested a new approach that uses community‐level data as a critical input for the network reconstruction process. This method focuses on directly predicting interspecies metabolic interactions in a community, when axenic information is insufficient. We validated our method through the case study of a bacterial photoautotroph–heterotroph consortium that was used to provide data needed for a community‐level metabolic network reconstruction. Resulting simulations provided experimentally validated predictions of how a photoautotrophic cyanobacterium supports the growth of an obligate heterotrophic species by providing organic carbon and nitrogen sources. J. Cell. Physiol. 231: 2339–2345, 2016. © 2016 The Authors. *Journal of Cellular Physiology* Published by Wiley Periodicals, Inc.

The role of microbial communities has been increasingly recognized in a multitude of scientific disciplines, including: soil ecology, environmental engineering, agriculture, food safety, and human health. Researchers have been keen to reveal the organizational principles in microbial communities and to predict their response to environmental cues, which requires a mechanistic understanding of interspecies interactions. Such understandings are also essential for designing and engineering microbial ecosystems for controllable outputs (Bernstein and Carlson, 2012; Lindemann et al., 2016). Advancements in multi‐omics analyses have significantly expanded the volume of biological data at hand, as well as our collective biological knowledge. However, experimental determination of diverse forms of microbial interactions in a community still remains a challenge. As a complementary tool, metabolic network analysis provides comprehensive predictions that can serve as ab initio hypotheses on cross‐species metabolite exchanges (Song et al., 2014; Biggs et al., 2015; Cardona et al., 2016).

Reconstruction of reliable microbial community networks is a challenging task. Even in the single‐species case, genome‐scale metabolic network reconstruction is an iterative process that takes a substantial period of time (Thiele and Palsson, 2010). Community modeling is a more challenging process due to the increased complexities involving interacting, non‐independent species. A conventional practice to build community metabolic networks focuses on the reconstruction of high‐quality individual networks so that their combination provides quantitative predictions of metabolic interactions and community behaviors (Shoaie et al., 2015). This approach becomes ineffective; however, if sufficient data required for curating individual networks are not available. This is often the case with environmental communities whose member species are not axenically cultivable or do not grow on defined media, for which we may lack high‐quality complete genome sequences.

To address this issue, we proposed an approach that directly refines metabolic networks at a community level. Community metabolic network reconstruction is primarily driven by a community‐level data, which is readily collectible even when member species are not isolable or individually cultivable. For testing the proposed approach, we considered a binary consortium composed of a *Thermosynechococcus elongatus* BP‐1 and *Meiothermus ruber* Strain A (hereafter *T. elongatus* and *M. ruber*, respectively) as a model photoautotroph–heterotroph consortium. We evaluated the qualities of resulting networks with a focus on automated reconstruction and refinement of draft networks. For this purpose, we used the DOE Systems Biology Knowledgebase (KBase) platform (www.kbase.us). Below, we describe reconstruction workflows for single species and extension of such protocols to the building of models of microbial communities, using our case study for illustration.

## Protocols of network reconstruction from single genomes

Current sequencing methods are unable to read a whole genome at a time, so all sequencing protocols first shear DNA into smaller fragments that the sequencer can read. To reassemble these fragments, many copies of the genome must be fragmented. That way, the shreds from one copy might overlap with the shreds from another copy so that the original DNA sequence can be reconstructed (Lander and Waterman, 1988). These contiguous stretches of overlapping fragments are called contigs. If the sequencing is deep enough, then these contigs can be assembled into one or more scaffolds that cover the full genome. Next, we need to know where genes are located and what their functions are. The most important genes to identify for a metabolic reconstruction are those that function as enzymes and transporters. The set of all reactions catalyzed by these enzymes comprises the intracellular metabolic network, and the transported substrates define the interface with the extracellular environment. The final step—formulation of a biomass synthesis equation—creates a testable metabolic model. If the metabolic network can produce all the essential compounds necessary for biomass from a set of extracellular metabolites, this is a prediction that the organism can grow in that nutrient condition. If we remove a reaction or transporter from the network, and it is no longer capable of producing biomass, then this is a prediction that the gene encoding that function is essential. By proceeding in this manner, metabolic network predictions of biomass production can be validated against growth or no‐growth phenotype observations. Procedures for network reconstruction of single genomes were summarized in Table S1.

## Strategies for community network construction

Community networks can be constructed from individual species’ genomes in many different forms. At the simplest level, we can take a mixed‐bag (or gene‐soup) approach by treating a microbial community as a single supra‐organism (Abubucker et al., 2012; Faria et al., 2016). The metabolic pathways and transmembrane transport reactions, resulting from all members, are combined by ignoring species boundaries. The mixed‐bag network has one cytosolic compartment and one extracellular compartment, analogous to a single species prokaryotic network. The primary usage of the mixed‐bag approach is to analyze environment‐community interactions. Construction of a mixed‐bag network requires the full genome sequences of all member species or a deep metagenome sequence. The growth conditions of the microbial community are also useful but species level resolution is not required.

**Figure 1:**
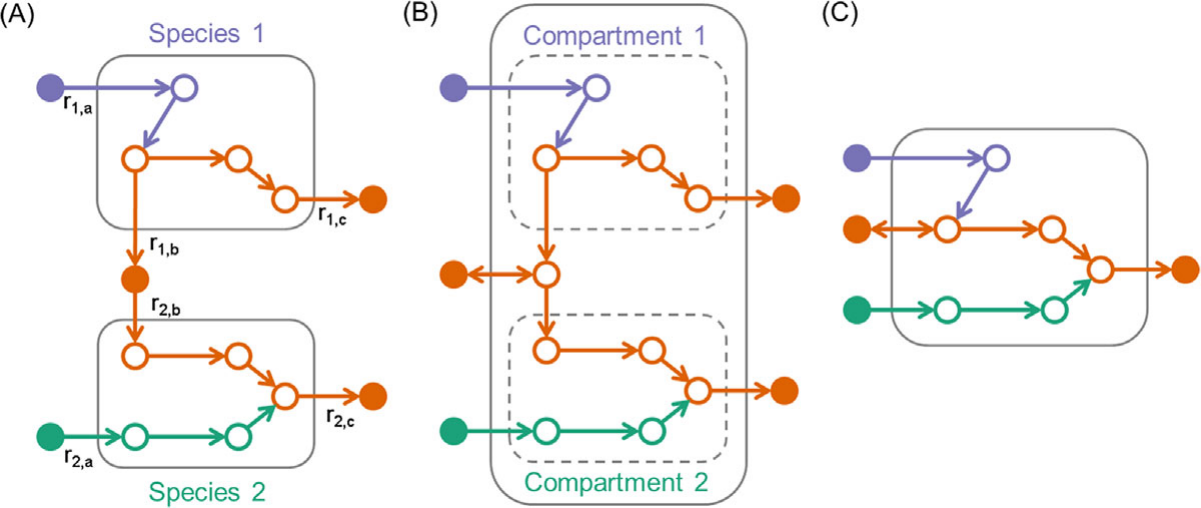
Alternative strategies for building community metabolic models for a binary consortium: (A) multi‐species dynamic modeling, (B) compartmentalized network modeling, (C) mixed‐bag network modeling. Colors represent metabolic pathways that are specifically associated with species 1 (purple) and species 2 (green), and that are common in both organisms (orange). Solid and open circles indicate extracellular and intracellular metabolites; solid line boxes represent compartments outside of which the quasi‐steady state assumption may not hold, and dashed line boxes represent compartments outside of which the steady‐state assumption continues to hold. In A, r_1_'s and r_2_'s denote exchange rates that are kinetically modeled.

Prediction of cross‐species metabolic interactions requires species‐resolved network modeling. This can be achieved by treating species networks as compartments of a community, the structure of which is similar to those of eukaryotic metabolic networks where inter‐ and intra‐compartmental activities are assumed to be in a quasi‐steady state. To predict non‐steady‐state microbial interactions, we require multi‐species dynamic models that account for kinetics of nutrient uptake and metabolite production for individual species. By overlaying species‐resolved kinetic expressions we can interrogate the dynamic interplay between members in a community. Multi‐species metabolic modeling (for a compartmentalized or kinetically connected model) starts with the construction of metabolic models for the individual species comprising the community. The methods of obtaining species‐resolved networks depend on the nature of the microbial community involved. For a defined community composed of isolated and cultivable microbes, we can use a single‐organism pipeline like the ModelSEED (Henry et al., 2010), RAVEN (Agren et al., 2013), or COBRA (Thiele and Palsson, 2010) to produce a separate model of each species. In contrast, for an undefined community derived from natural environment, we need a process to build species‐level metabolic models from the metagenomics data by separating out the assembled contigs into species‐level bins and subsequently performing gene calling, annotation, and model building on each bin. In Figure 1, we illustrated the three alternative approaches for building microbial community metabolic models described in this section.

## A binary consortium to model photoautotroph–heterotroph communities

With a focus on steady state analyses (i.e., methods shown in Fig. 1B and C), we will explore the advantages and disadvantages of the various strategies for constructing microbial community models through the use of a model microbial consortium, comprised of the photoautotrophic cyanobacterium *T. elongatus* and an obligate aerobic heterotroph *M. ruber*. Cyanobacteria are essential contributors to primary productivity in natural ecosystems and are frequently associated with dependent populations of heterotrophic bacteria. These phototrophic‐heterotrophic communities are of keen interest to biologists seeking to uncover mechanisms of interaction that drive global biogeochemical cycles (e.g., involving C, N, and O_2_). They are also attractive bioprocess hosts because of their inherent robustness, diverse metabolisms, and fast growth rates. We selected these specific taxa to demonstrate community modeling because of the obligate dependency of *M. ruber* on *T. elongatus*, when cultured under autotrophic conditions. Depending on the environment, *M. ruber* relies upon cyanobacterial derived organic‐carbon, fixed nitrogen, vitamins, and O_2_.

*T. elongatus* BP‐1 is a thermophilic, unicellular cyanobacterium that has been studied extensively (Onai et al., 2004; Zhang et al., 2005; Arai and Kino, 2008; Abed et al., 2009; Eberly and Ely, 2012). Hence, it is a model cyanobacterium and has a completely sequenced and annotated genome (Nakamura et al., 2002). *T. elongatus* was isolated from a cyanobacterial mat environment near Beppu, Japan (Yamaoka et al., 1978). *M. ruber* Strain A is an aerobic, heterotrophic, thermophile that was isolated from an enrichment culture originally sampled from the cyanobacterial mat inhabiting Octopus Spring in Yellowstone National Park (WY, USA) (Thiel et al., 2015). It shares 98.6% nucleotide identity to the 16S rRNA gene sequence of *M. ruber* DSM 1279 (Loginova et al., 1984) and displays strong functional relatedness with regard to central carbon and energy metabolism genes (Thiel et al., 2015). Similar to *M. ruber* DSM 1279, Strain A lacks the nitrate assimilatory pathway. Hence, it depends upon reduced N‐sources produced by *T. elongatus* when growing in an environment that is depleted of fixed/reduced nitrogen.

## Reconstruction of single species metabolic models using the KBase platform

Before we can construct a community model of *T. elongatus* and *M. ruber*, we first need to construct individual metabolic models for each of these species. The KBase platform offers two alternative pipelines for the generation and refinement of metabolic models from sequence data as described in the following. One pipeline enables the direct reconstruction of a new draft model from a genome sequence, consisting of four steps: (i) import of the genome into KBase; (ii) structural and functional annotation of the genome using the RAST annotation algorithm (Aziz et al., 2008); (iii) reconstruction of a draft metabolic model using the ModelSEED algorithm (Henry et al., 2010); and (iv) gapfilling the model on a specified growth condition using optimization‐based approaches (Dreyfuss et al., 2013; Latendresse, 2014). The final gapfilling step can be performed iteratively on multiple media conditions if a capacity for growth under multiple conditions has been confirmed experimentally.

We applied this pipeline to build a model of the heterotrophic member from our example community, *M. ruber* (https://narrative.kbase.us/narrative/ws.13807.obj.1). We created three versions of our *M. ruber* model: (i) an ungapfilled version (*d*Mr729); (ii) a version gapfilled on a rich LB media in which *M. ruber* is known to grow (*lb*Mr729); and (iii) a version gapfilled on glucose minimal media (*mm*Mr729), on which it is known not to grow.

Table I shows the number of reactions, genes, transporters, transportable metabolites, and gapfilled reactions contained in each model. Flux balance analysis (FBA) of the model gapfilled on LB media predicted that this species lacks a capacity to synthesize several vitamins/cofactors (niacin, pantothenate, riboflavin, and heme) and amino acids (proline, valine, isoleucine, lysine, and histidine). These results are largely (albeit not totally) consistent with experimental data on the nutrients required for culturing *M. ruber* Strain A. While this result is encouraging, it is also well‐established that draft models constructed directly from genome sequence data typically require substantial curation before they can be truly predictive (Thiele and Palsson, 2010).

**Table I:** Statistics on all individual and community metabolic models constructed

Model	Reactions	Genes	Transporters	Transported metabolites	Gapfilled reactions
*d*Mr729	1163	729	68	61	0
*lb*Mr729	1253	729	76	71	95
*mm*Mr729	1257	729	71	65	98
*d*Te583	889	583	88	86	0
*a*Te583	917	583	89	87	28
*mb*MrTe1312	1707	1312	124	93	74
*pre*MrTe1312	2174	1312	160	91	126
*mix*MrTe1312	2175	1312	168	100	128
*post*MrTe1312	2168	1312	164	98	123

Fortunately, it is not always necessary to start from scratch when building a model from a genome sequence. KBase offers an alternative pipeline in cases where a curated model already exists for a close relative of the genome of interest. This pipeline consists of five steps: (i) import the genome; (ii) annotate the genome; (iii) import a curated model and genome of a closely related species; (iii) perform bi‐directional all‐versus‐all BLAST comparison of the genes in the primary genome and the well‐curated relative; (iv) propagate the curated model to the genome of interest based on bi‐directional best gene hits; and (v) optionally gapfill the model on a specified growth conditions using optimization‐based approaches. We applied this second pipeline in KBase to build a model of *T. elongatus* based on the previously published *i*JN678 model of *Synechocystis sp. PCC 6803* (Nogales et al., 2012) (https://narrative.kbase.us/narrative/ws.13806.obj.1). As it is known that *T. elongatus* is autotrophic, we only generated two versions of this model (Table I): (i) an ungapfilled model (*d*Te583) and (ii) a model gapfilled on autotrophic media (*a*Te583).

## Reconstruction of consortium metabolic models using the KBase platform

Now that we have single species models constructed for both of the members of our example consortium, we can combine these models into either a mixed‐bag community model (Fig. 1C) or a compartmentalized community model (Fig. 1B). As before, we applied the KBase platform to build both model types. When constructing a mixed‐bag model, the reactions from each single‐species model are merged together into a single intracellular compartment. Gene associations are preserved from the original models, and they are merged in the cases where the same reaction appears in multiple models. The biomass objective functions from all individual models are combined together into a single consortium biomass objective function by summing the stoichiometric coefficients from each individual biomass reaction, and rescaling the coefficients by the number of models being combined. Like individual models in KBase, mixed‐bag models can be gapfilled in a specified growth condition. We applied this capability in KBase to combine our ungapfilled *M. ruber* and *T. elongatus* models into a mixed bag model (https://narrative.kbase.us/narrative/ws.13838.obj.1), which we subsequently gapfilled in autotrophic media. We call this model *mb*MrTe1312 (Table I). Here we see that the mixed‐bag model has fewer total reactions than the sum of the two constituent models. Additionally, the mixed‐bag model required more gapfilled reactions than either single model, but fewer than both models combined.

Next we applied tools in KBase to construct a compartmentalized model of our model consortium. When constructing a compartmentalized community model in KBase, the two individual models are still combined into a single model, but in this case, the reactions from each individual model are integrated into distinct cellular compartments in the new consortium model. These two compartments share a single common extracellular compartment that permits the exchange of metabolites between the species models. As with all models in KBase, compartmentalized community models can be gapfilled. In this case, gapfilling is distinctive in that the entire database of candidate reactions is added to each separate species compartment in the consortium model, meaning the number of candidate reactions considered by the gapfilling scales with the number of species models combined in the consortium model, which in turn results in a greater complexity to the gapfilling formulation and a longer run‐time in finding an optimal solution. However, this formulation offers a decisive advantage over gapfilling of individual models, as the algorithm now considers the possibility that metabolic functions may be delegated among the species comprising the consortium, and as such, will propose solutions that involve interactions between species in the cases where such interactions result in the greatest parsimony. This is particularly valuable for gapfilling models of species that cannot be grown in isolation, because the only phenotypic data available for these species involves multiple organisms operating in concert. We now explore the impact of applying a range of gapfilling strategies when constructing a compartmentalized model of a microbial consortium.

## Alternative gapfilling strategies in reconstruction of consortium metabolic models

**Figure 2:**
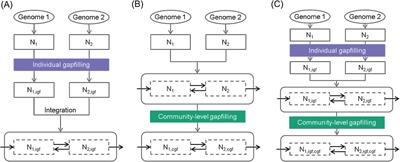
Alternative gapfilling strategies when constructing compartmentalized consortium metabolic models: (A) individual gapfilling (igf), (B) community‐level gapfilling (cgf), (C) combination of A and B. N_1_ and N_2_ denote draft networks of species 1 and 2, respectively.

Because it is possible to gapfill models on a variety of growth conditions, and because it is possible to gapfill models both before and after they have been merged together into a compartmentalized consortium model, we are presented with a wide range of potential strategies for constructing a consortium model. We summarize the many potential options with three fundamental alternative approaches: (i) pre‐gapfilling individual models on minimal media prior to merging into a community model (Fig. 2A); (ii) merge ungapfilled individual models into a community model, then gapfill the community model (Fig. 2B); or (iii) pre‐gapfill individual models on rich media, merge into a community model, then post gapfill the community model (Fig. 2C).

**Figure 3:**
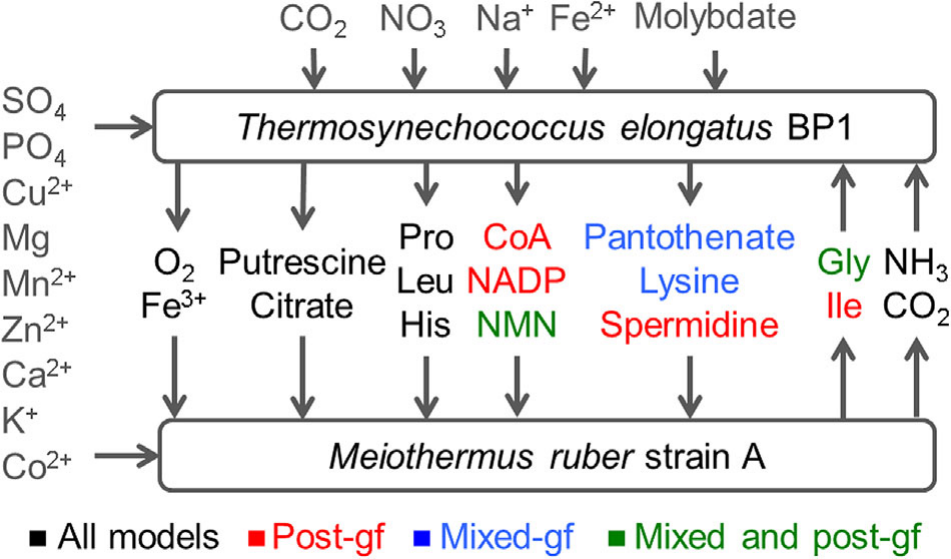
Interactions between *M. ruber* and *T. elongatus* predicted by consortium models.

We applied all three alternative strategies in KBase (https://narrative.kbase.us/narrative/ws.13838.obj.1), resulting in the development of three alternative compartmentalized models of our *M. ruber* and *T. elongatus* consortium: (i) pre‐gapfilled *pre*MrTe1312; (ii) post‐gapfilled *post*MrTe1312; and (iii) gapfilled *mix*MrTe1312 (Table II). We then simulated the growth of these three consortium models in autotrophic media using FBA and identified the interspecies interactions predicted by each model (Fig. 3). As expected, the *pre*MrTe1312 model predicts the fewest interactions with only 9 metabolites exchanged, while *post*MrTe1312 predicts the most interactions, with 15 metabolites exchanged. The *mix*MrTe1312 model splits the difference, predicting 13 metabolites exchanged. Thus, we see that typically more interactions are predicted when more of the gapfilling is conducted after the individual models have been merged into a community model. This trend occurs because gapfilling conducted on merged community models will consider solutions that involve trophic interactions between species, and where such trophic interactions may actually be occurring, these solutions will typically be lower cost than the non‐interactive alternative of adding all biosynthetic pathways to constituent species. We also highlight that the pre‐gapfilling approach may be ineffective in a typical situation where some of the members are cultivable only on rich media, because choosing to gapfill their networks on rich media alone may be insufficient to identify microbial interactions that enable community growth.

**Table II:** Consistency between reaction flux and gene expression in various model versions

	+Flux, +Exp (%)	−Flux, −Exp (%)	+Flux, −Exp (%)	−Flux, +Exp (%)	Accuracy for active genes (%)	Accuracy for inactive genes (%)	Overall accuracy (%)
*mm*Mr729	16.1	37.0	17.5	29.5	35.3	67.9	53.1
*lb*Mr729	25.5	15.9	5.6	53.0	32.5	74.0	41.4
*a*Te583	54.5	14.9	9.4	21.2	72.0	61.2	69.4
*mb*MrTe1312	20.5	35.2	20.4	23.9	46.2	63.3	55.7
*pre*MrTe1312	17.5	37.1	25.0	20.4	46.2	59.7	54.6
*mix*MrTe1312	17.4	37.0	25.2	20.5	45.9	59.5	54.4
*post*MrTe1312	17.6	37.1	25.0	20.4	46.3	59.7	54.6

Of our three compartmentalized community models, the interactions predicted by the *post*MrTe1312 model also best match our experimental understanding of the dependencies between *M. ruber* and *T. elongatus* growing on autotrophic media, although it is worth noting that the pantothenate exchange predicted by the *mix*MrTe1312 is more physiologically reasonable than the exchange of CoA predicted by *post*MrTe1312.

## Evaluation of community networks in comparison with gene expression profiles

In addition to evaluating our models based on the number and type of gapfilled reactions and the predicted trophic interactions, we can also evaluate the capacity of our models to accurately predict which pathways will be active in our organisms while they are growing together in a consortium. To accomplish this, we gathered RNA‐seq data for our *M. ruber* and *T. elongatus* consortium growing in autotrophic conditions. We mapped the reads to our individual *M. ruber* and *T. elongatus* genes and loaded the expression profile into KBase. We also called each gene “active” or “inactive” based on its expression level (see the next section). Finally, we ran transcriptomic FBA (Seaver et al., 2015) to fit the flux predictions made by our models to gene activity computed from our RNA‐seq data. This approach attempts to force reactions associated with inactive genes off, while forcing reactions associated with active genes on, without preventing the model from producing biomass. We thus compared our models’ flux predictions with active/inactive gene activity based on gene expression data for enzymes catalyzing reactions in our models.

We ran this analysis on our two gapfilled *M. ruber* models: *mm*Mr729 and *lb*Mr729 (Table II). The *mm*Mr729 model performed better at predicting active reactions, correctly predicting 35% of reactions associated with actively expressed genes as active (vs. 33% predicted by the model in LB media). However, the *lb*Mr729 model performed much better at predicting inactive reactions, correctly predicting 74% of reactions associated with poorly expressed genes as inactive (vs. 68% predicted by the model in glucose minimal media). These results show that minimal media activates too much of the metabolic network, while LB media activates too little. However, these results also show that both models suffer from inaccuracies in their capacity to produce flux profiles that are consistent with observed patterns in gene expression. Much of this inaccuracy is likely due to comparing the flux of a single species model with the gene expression data generated from a community in which it is metabolically dependent on another member. However, some inaccuracy will typically be the case from draft models constructed directly from genome sequence, which was unavoidable in the case of *M. ruber*, as no published curated model currently exists for this species or any close relatives of this species.

We conducted the same analysis on our *T. elongatus a*Te583. For this model, we saw significantly improved accuracy compared with the *M. ruber* model (Table II). Our *T. elongatus* model correctly predicted 72% of reactions associated with highly expressed genes as active; and it correctly predict 61% of reactions associated with poorly expressed genes as inactive. This result clearly shows the value of starting analysis with a curated model whenever one is available. In this case, the gene expression data are also more directly comparable to the case of a single genome model as *T. elongatus* is not dependent upon *M. ruber* and can grow in isolation on the media condition used to generate the expression profile.

**Figure 4:**
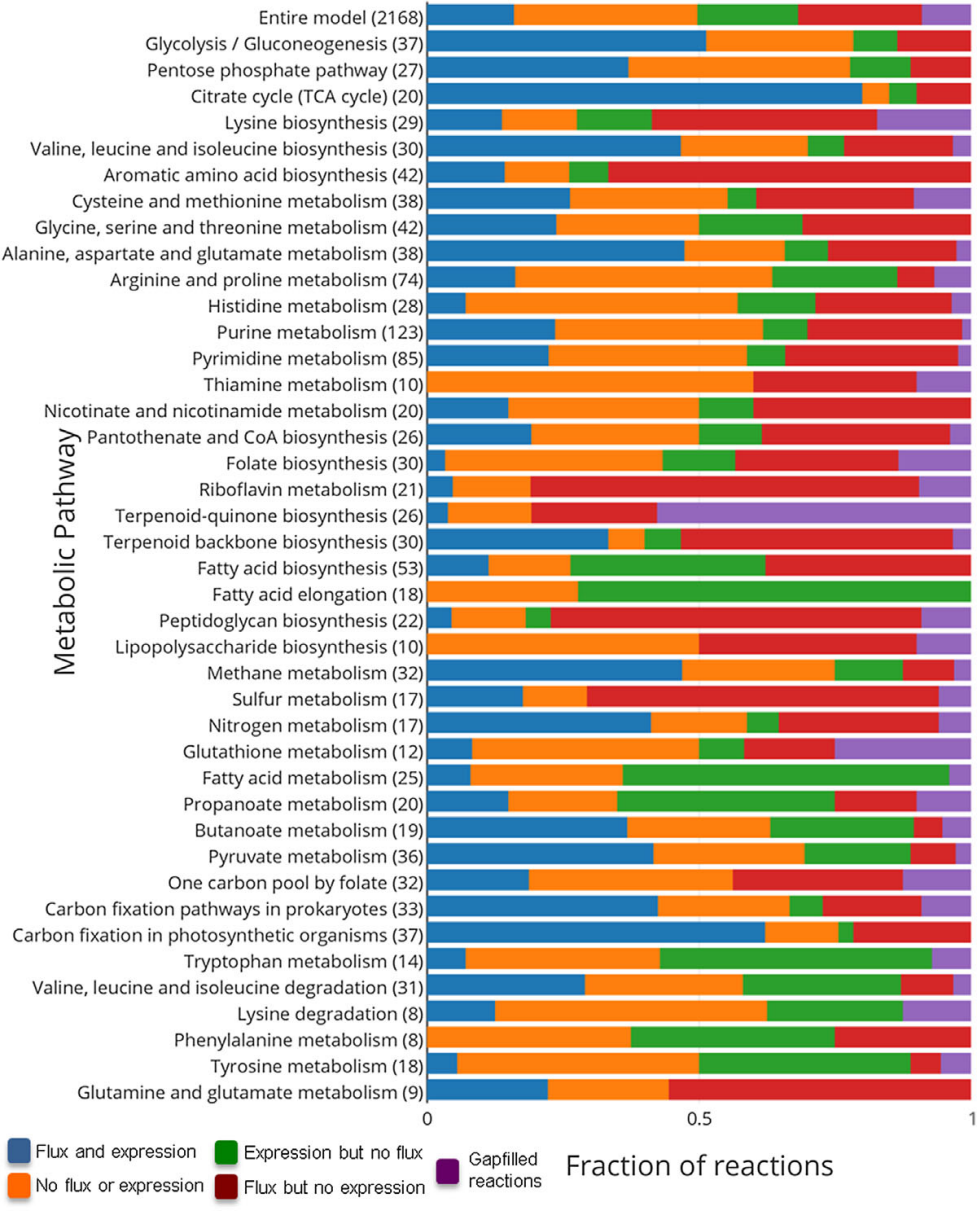
Comparison of flux and expression for post‐gapfilled consortium model. Here, we show the extent of agreement between the reaction activity predicted by FBA and gene expression derived from RNA‐seq data for the consortium *post*MrTe1312 model. Blue bars denote reactions with both flux and expression, while orange bars denote reactions without flux or expression. Green bars denote reactions with expression but no flux, while red bars denote flux but no expression. Finally, purple bars denote gapfilled reactions active in each pathway. The number of reactions in each metabolic pathway is shown in the parenthesis.

Finally, we ran the analysis with our gapfilled mixed‐bag model, as well as all three compartmentalized models (Table II). The mixed‐bag model performed the best of our community models, but this model type provides no information on potential interactions among the species in our consortium. Among our compartmentalized models, the *post*MrTe1312 performed the best, although all three compartmentalized models produced very similar levels of performance. We can also see a pathway‐by‐pathway breakdown of the agreement between model flux and expression data (Fig. 4). This analysis shows that the riboflavin and quinone pathways showed the poorest agreement, indicating potential flaws in the biomass equation of the *M. ruber* model.

## Calling genes “on” or “off” based on expression level

In order to compare the reaction activity predicted by our models with our gene expression data, it was necessary to classify genes as either “on” or “off” based on their level of expression in our RNA‐seq data. To accomplish this, we started with a list of 80 functional roles from the SEED that we have identified as universally active, largely from translation and transcription. We then determined the genes in our genomes that have been annotated with these functions, and obtained the expression values associated with these genes. We ranked these genes based on their expression values, and selected the expression value located at the 10th lowest percentile as the threshold for calling genes “on” or “off.” Genes with expression values higher than this threshold are called “on,” and genes with expression values lower than this threshold are called “off.”

## Summary and future directions

Here, we provided a new approach that uses community‐level data for microbial community network reconstruction, and we demonstrated tools that implement this approach in a user‐friendly manner in the DOE Systems Biology Knowledgebase. Development of this approach was motivated by: (i) community data provides key information on microbial interactions, which are not necessarily obtainable from axenic cultures and (ii) the conventional approaches that require high‐quality individual networks are ineffective when member species in a community cannot be sufficiently characterized in isolation. In the case study of a binary consortium, metabolic networks reconstructed using this method led to predictions of phototroph–heterotroph interactions that are consistent with experimental data. Manual curation is a necessary next step for more accurate predictions. Further developments using KBase are in progress to extend the proposed method to more complex communities beyond simple binary consortia.

## Supporting information

Additional supporting information may be found in the online version of this article at the publisher's web‐site.

**Table S1**. Network reconstruction protocol for individual genomes.
