# Supplementary material for: Microbial Community Metabolic Modeling: A Community Data‐Driven Network Reconstruction
Source: J Cell Physiol. 2016 Jun 2;231(11):2339–45. doi: 10.1002/jcp.25428 (PMC5132105; doi:10.1002/jcp.25428)
Supplement: Supplementary file 1 — Table S1. Network reconstruction protocol for individual genomes. [file JCP-231-2339-s001.docx]

**Table S1.** Network reconstruction protocol for individual genomes.

| Protocol step | Evidence | Prediction |
| --- | --- | --- |
| Contig assembly | Short read sequences | Contiguous overlapping reads (Contigs) |
| Genome assembly | Contigs, paired-end reads, mate-pair reads | Ordered contigs separated by NNNs (Scaffold) |
| Gene structure annotation | Genome assembly, transcriptome data, foreign proteins | Location of genes on assembly |
| Protein function curation | Gene structure predictions,  Reference protein function database | Enzymes, transporters and protein complexes |
| Draft network reconstruction | Enzyme and transporter predictions  Universal pathway database | Draft metabolic network |
| Phenotype-directed curation | Phenotype observations of wild-type and mutants in defined media  Expression data of wild-type and mutants in defined media | Curated metabolic network |
